# COVID-19 and vaccination in hereditary angioedema: Single center experience^☆^

**DOI:** 10.1016/j.waojou.2024.100892

**Published:** 2024-03-23

**Authors:** Öner Özdemir, Ümmügülsüm Dikici

**Affiliations:** Division of Allergy and Immunology, Department of Pediatrics, Sakarya University Faculty of Medicine, Research and Training Hospital of Sakarya University, Sakarya, Türkiye

**Keywords:** Hereditary angioedema, COVID-19, SARS-CoV-2, Vaccination

## Abstract

Like many microbial agents, Severe acute respiratory syndrome coronavirus 2 (SARS-CoV-2) and its vaccination may increase the frequency and/or severity of attacks. We aimed to observe/evaluate patients with hereditary angioedema (HAE) followed by Sakarya University Research/Training Hospital pediatric allergy unit, Sakarya, Türkiye, for the effects of SARS-CoV-2 infection and COVID-19 vaccination. Ten HAE patients—3 males and 7 females—were evaluated retrospectively. Their mean age was 31.80 ± 19.15 (min. 12 – max. 66) years. Four of 10 patients were diagnosed with type 1 HAE and 6 with type 2 HAE. Two out of 6 patients (mother and daughter) diagnosed with type 2 HAE had angioedema attacks during COVID-19 disease. Six out of 10 HAE patients received different COVID-19 vaccines available in Türkiye up to third and fourth doses. There was no increase in COVID-19 vaccine-related attacks during, after 72 hours, and up to the year after vaccination. As a result, we consider it safe to administer inactivated and/or mRNA vaccines in our patients with HAE. In addition, catching SARS-CoV-2 infection was not always associated with disease exacerbation or activation.

To the Editor,

Severe acute respiratory syndrome coronavirus 2 (SARS-CoV-2) enters host cells via angiotensin-converting enzyme 2 (ACE2) and diminishes ACE2 expression, essential for bradykinin metabolism. Consequently, the accumulation of bradykinin occurs, which affects bradykinin receptors and causes vasodilation.^1^^,^^2^ Like many microbial agents, SARS-CoV-2 and its vaccination may increase the frequency and/or severity of hereditary angioedema (HAE) attacks.^3^^,^^4^ This study aimed to observe/evaluate HAE patients followed by Sakarya University Research/Training Hospital pediatric allergy unit, Sakarya, Türkiye, for the effects of SARS-CoV-2 infection and COVID-19 vaccination.

Ten patients with HAE—3 males and 7 females—were evaluated retrospectively (Table 1) with a mean age of 31.80 ± 19.15 (min. 12 – max. 66) years (4 type 1 HAE and 6 type 2 HAE).^5^ (Non-interventional ethics committee approval number: E-71522473-050.01.04-2581-22-192.) The enrolled subjects underwent complement testing (C4, C1-INH antigen, and functional C1-INH). C4 and C1-INH antigen were measured using the nephelometric methods. C1-INH function was measured using a chromogenic method. If the laboratory tests were abnormal, the enrolled subjects were questioned on clinical manifestations and scheduled for a follow-up visit.

**Table 1:** Hereditary angioedema patients followed in our center during SARS-CoV-2 infection and vaccination.

Case #	Gender	Age	Types	On-demand / Attack Treatment	Prophylaxis Treatment	Attack during COVID-19	Type and number of vaccines	Post vaccine attack
Case 1	F	33	Type 2	Icatibant / pd C1-INH	–	Abdominal attack	No vaccine application	–
Case 2	M	12	Type 2	Icatibant / pd C1-INH	Tranexamic acid/	Not caught	No vaccine application	–
Case 3	F	19	Type 2	Icatibant / pd C1-INH	–	Not caught	2 Sinovac, 1 BioNTech	No
Case 4	M	66	Type 2	Icatibant / pd C1-INH	Danazol	Not caught	2 Sinovac, 1 BioNTech	No
Case 5	F	32	Type 2	Icatibant / pd C1-INH	–	Not caught	2 Sinovac, 2 BioNTech	No
Case 6	F	17	Type 1	Icatibant / pd C1-INH	–	No attack	No vaccine application	–
Case 7	M	50	Type 1	Icatibant / pd C1-INH	Danazol	No attack	2 Sinovac	No
Case 8	F	12	Type 1	Icatibant / pd C1-INH	–	No attack	No vaccine application	–
Case 9	F	22	Type 1	Icatibant / pd C1-INH	–	Not caught	1 Sinovac	No
Case 10	F	55	Type 2	Icatibant / pd C1-INH	Tranexamic acid/Danazol	Abdominal, extremity, and laryngeal attack	1 Turkovac	No

pd C1-INH: plasma derived C1-INH concentrate

Five out of 10 HAE patients have had a SARS-CoV-2 infection. The 4 patients who contracted SARS-CoV-2 had a mild illness. One (mother with type 2 HAE) had a mild-to-moderate disease due to her older age and co-morbidity such as hypertension. These patients had taken no other medication other than favipiravir, which was routinely used in the country for 5 days at the time. Two out of 5 patients (mother and her daughter) diagnosed with type 2 HAE had angioedema attacks during COVID-19 disease. While the mother had abdominal, extremity, and laryngeal attacks, her daughter had an abdominal attack. C1-INH concentrate was administered in repeated doses for the patients. Two patients with infection-related HAE attacks were evaluated with angioedema activity score (AAS) −7 scoring (Table 2). Despite the use of C1-INH replacement therapies during the attack, the score of the mother with type 2 HAE increased from 0 before the attack to 45 and that of her daughter to 60. However, another family (father and 2 daughters) diagnosed with type 1 HAE also had COVID-19 disease without any attack. Our 3 patients (Type 1 HAE) had no attacks, and 2/6 type 2 HAE patients had an attack during infection, which might be associated with the SARS-CoV-2 variant and a more significant C1-INH functional deficiency in these patients.^5^ We are unsure whether there is a difference between HAE types for getting SARS-CoV-2 infection since HAE types occur due to a different pathophysiology.^1^

**Table 2:** Angioedema activity score (AAS)-7 of our hereditary angioedema patients followed in our center during SARS-CoV-2 infection and COVID-19 vaccination.

Case #	Gender	Age	Types	Pre- COVID-19 AAS	AAS post- COVID-19	AAS pre-vaccine	AAS post-vaccine
Case 1	F	33	Type 2	0	60	No vaccine application	No vaccine application
Case 2	M	12	Type 2	Not caught	Not caught	No vaccine application	No vaccine application
Case 3	F	19	Type 2	Not caught	Not caught	0	0
Case 4	M	66	Type 2	Not caught	Not caught	0	0
Case 5	F	32	Type 2	Not caught	Not caught	0	0
Case 6	F	17	Type 1	0	0	No vaccine application	No vaccine application
Case 7	M	50	Type 1	0	0	0	0
Case 8	F	12	Type 1	0	0	No vaccine application	No vaccine application
Case 9	F	22	Type 1	Not caught	Not caught	0	0
Case 10	F	55	Type 2	0	45	0	0

AAS -7: angioedema activity score for daily scoring for 7 days

Six out of our 10 HAE patients received various COVID-19 vaccines [mRNA: BioNTech® and inactivated: Sinovac® (CoronaVac®), and Turkovac®] available in Türkiye up to third and fourth doses. There was no increase in vaccine-related attacks during and after 72 hours and up to the year after vaccination.^5^ After vaccination, none of the patients showed an increase in AAS-7 scoring compared to before. COVID-19 vaccine-related angioedema attack development was previously described in patients with HAE. Although a slight increase in attacks has been reported, some study groups had no intense exacerbations.^1^^,^^6^ In a study, 5/31 patients (16.1%) experienced angioedema attacks 72 hours after the first dose.^7^ Another study demonstrated that one angioedema attack (1.2%) was found to be associated with the vaccination in a total of 86 doses. There was a slight increase in the typical amount of attacks in the year after vaccination.^4^ The authors found that 7.4% of vaccine doses intensified HAE attacks after the first vaccinations.^6^ Fijen et al,^8^ and Ieven et al,^9^ respectively, demonstrated 10% and 6.3% percentages of the first 2 vaccine doses followed by an HAE attack.

The possible interaction between COVID-19 vaccinations and long-term prophylaxis (LTP) agents, eg, attenuated androgens / Danazol, should be briefly mentioned in these patients. Danazol was used irregularly as LTP in a couple of our 10 HAE patients. Two of our 10 HAE patients used tranexamic acid as LTP as well. Furthermore, some of our HAE patients needed on-demand utilization of Icatibant and plasma-derived C1-INH concentrate. There have been concerns about the use of attenuated androgens, eg, Danazol, owing to its capacity to prevent the antiviral immune response to SARS-CoV-2 or modify the expression of the TMPRSS2 (Transmembrane Serine Protease 2) receptor, the SARS-CoV-2 utilizes.^2^ Some authors suggested putting HAE patients on short-term prophylaxis before vaccination.^4^ We did not observe any specific adverse effect linked with LTP (eg, Danazol or tranexamic acid) utilization during or after SARS-CoV-2 infection and vaccination, which is similar to some literature.^7^

As a result, it is good to see that it is safe to administer inactivated and/or mRNA vaccines in our patients. In addition, we have also observed that catching SARS-CoV-2 infection does not always lead to disease exacerbation or activation.

## Abbreviations

SARS-CoV-2, Severe acute respiratory syndrome coronavirus 2; ACE2, Angiotensin-converting enzyme 2; HAE, Hereditary angioedema; C1-INH, C1-inhibitor; C1-INH HAE, C1-inhibitor hereditary angioedema; LTP, long-term prophylaxis; TMPRSS2, Transmembrane Serine Protease 2

## Funding information

No funding sources.

## Availability of data and materials

The data that support the findings of this study are available from the corresponding author upon reasonable request.

## Authors' contributions

Dr. Özdemir wrote the article and Dr. Dikici assisted Dr. Özdemir.

## Ethics approval

Non-interventional ethics committee approval number: E-71522473-050.01.04-258122-192.

## Authors’ consent for publication

All authors approved the latest version of manuscript.

## Declaration of competing interest

No conflict of interest.
